# Accuracy of single ceramic crowns milled on a mobile digital dental laboratory after transportation over varying distances

**DOI:** 10.1016/j.jds.2024.11.031

**Published:** 2024-12-07

**Authors:** Hsuan Chen, Pey-Fen Tang, Shyh-Yuan Lee, Yuan-Min Lin

**Affiliations:** a Department of Dentistry, National Yang Ming Chiao Tung University, Taipei, Taiwan; b Department of Stomatology, Taipei Veterans General Hospital, Taipei, Taiwan; c Institute of Oral Tissue Engineering and Biomaterials, National Yang Ming Chiao Tung University, Taipei, Taiwan; d Oral Medicine Innovation Center, National Yang Ming Chiao Tung University, Taipei, Taiwan

**Keywords:** Accuracy, Single crown, Vibration, Mobile lab, CNC milling, Circuit dental service

## Introduction

Taiwan, as a small island, is among the most mountainous regions in the world. Approximately 70 % of the island is shrouded in densely forested mountains, featuring 286 peaks that rise above 3000 m. This remarkable landscape is a direct consequence of Taiwan's position on the border of Eurasian and Philippine Sea Plates, a factor that contributes to the ongoing increase in the number of peaks.

As a result, many areas in Taiwan are separated from cities by mountains, leaving residents in these regions facing significant barriers to regular dental care due to distance, limited transportation, and a shortage of dental professionals. This issue is particularly pronounced in Eastern Taiwan, where the region's elongated and narrow terrain exacerbates the uneven distribution of medical resources. The need for prosthodontic treatment is especially pressing, as 36.6 % of the population aged 65–74 has fewer than 20 natural teeth,^1^ a condition associated with compromised oral function and quality of life. Circuit dental services have been routinely operated throughout the island to alleviate transportation burdens for residents of remote areas. Circuit dental services treat over one hundred thousand patients annually, providing care such as oral hygiene instruction (OHI), ultrasound scaling, caries removal, fillings, and denture adjustments.^2^ However, these offerings remain limited and have yet to evolve significantly over the past few decades. As a result, patients requiring prosthodontic treatments, such as dentures or other oral rehabilitative procedures, are often underserved. The lack of such critical services highlights the urgent need to expand the scope of circuit dental care to meet the growing demand for prosthodontic treatment, particularly in remote and resource-limited regions like Eastern Taiwan.

Given this, the authors built a mobile digital dental laboratory designed to provide complete digital dental laboratory support in remote areas. Furthermore, the integration of modern technology such as a dental CNC (Computer Numerical Control) milling machine into mobile digital dental laboratory allow for high-precision restorations to be created on the spot. This reduces wait times for patients and streamlines the overall treatment process. Patients benefit from receiving high-quality dental care that is typically only available in established dental offices without having to travel long distances.

Dental milling machines are precise and delicate units that require routine calibration and extra care. It was generally believed that dental milling machine is not something that can be transported from one spot to another spot routinely, especially if they are not fully-wrapped with shock and vibration protection materials in a wooden crate.

Despite the above-mentioned transportation method is widely adopted by many manufacturers, unboxing, setup, and connecting the machine to the peripheral devices upon arrival is time-consuming and tedious. It also reduced the precious time for circuit dental services. Therefore, in the mobile digital dental lab we built, the dental milling machine was designed to firmly attached to the vehicle. To ensure that the dental milling machine after transportation can manufacture prosthesis which can satisfy the clinical needs, the accuracy of the milled prosthesis should be evaluated.

The objective of this study was to evaluate the accuracy of the milled crown by dental milling machine after transportation for various distance. The vibration of the dental milling was also recorded for reference.

## Materials and methods

**Figure 1:**
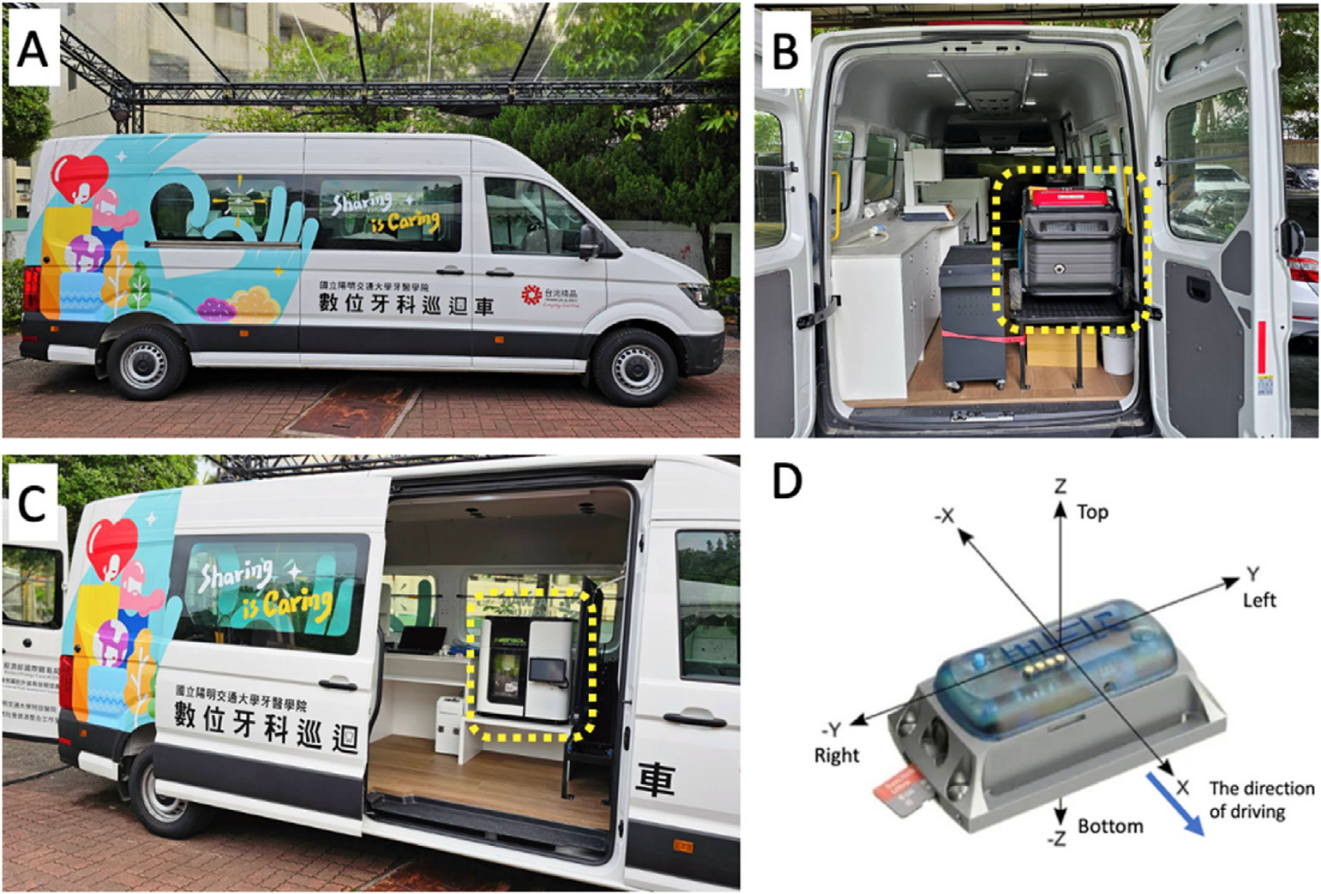
The exterior and equipment of the mobile digital dentistry lab: (A) Exterior view of the mobile digital dentistry lab. (B) Honda power generator (highlighted with a dashed line). (C) Dental milling machine (highlighted with a dashed line). Both the Honda power generator and the dental milling machine were securely mounted on a custom-made rack, which was firmly fixed to the floor of the load compartment for stability during operation and transport. (D) Orientation of the vibration sensor, firmly attached to the dental milling machine.

A mobile digital dental lab was designed and built using a Volkswagen Crafter (Volkswagen, Wolfsburg, Germany) as carrier. An Idensol 5XG five-axis dental milling machine (Toshin, Taichung, Taiwan) was mounted on the custom-made table which was firmly fixed to the floor of the load compartment (Fig. 1).

A Honda EU7000 power generator (Honda Motor Co., Ltd., Shizuoka-ken, Japan) was installed at the end of the load compartment. To facilitate the air circulation, a rear door was opened during the operation of the power generator, and two industrial fans were used to enhance the exhaust removal. Therefore, the mobile digital dental lab was self-powered, and no power cord was needed to connect to a wall socket.

**Figure 2:**
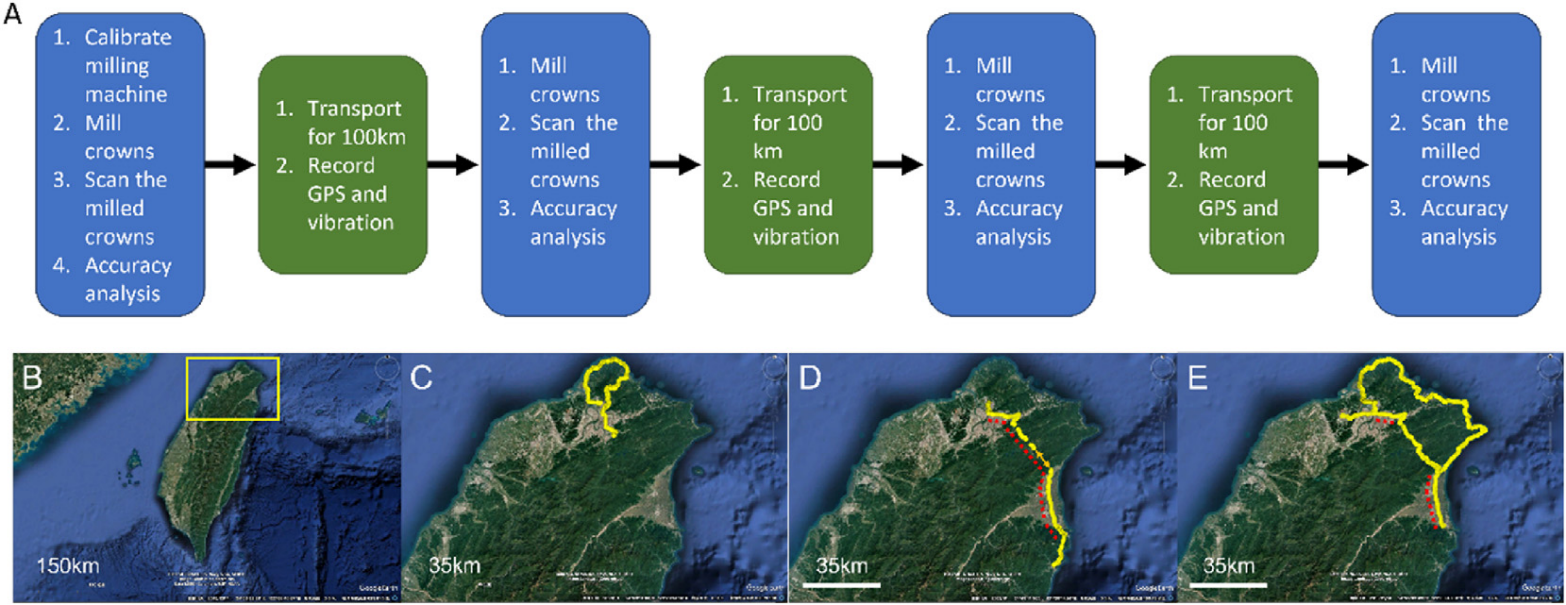
(A) The experimental procedure. Abbreviation: GPS (Global Positioning System). (B) Map of Taiwan. A closer view of the area highlighted in the yellow box is shown in panels C, D, and E. (C) 100 km route. (D) 200 km route, with the orange arrow indicating transportation through a tunnel where GPS data was not recorded. (E) 300 km route. In panels D and E, red dashed lines represent the highway or expressway portions of the routes.

In order to simulate the actual working conditions, we drove the mobile digital dental laboratory following three different driving routes before each batch of tests. The experimental procedure was performed as shown in Fig. 2A.

The driving routes included various types of roads, such as urban streets, mountainous roads, and highways, as illustrated in Fig. 2B and C. Three driving routes were selected with distances of 100 km, 200 km, and 300 km. Each route featured a combination of urban roads, highways, and tunnels to simulate diverse driving conditions. These routes were chosen based on the actual paths traveled by our team during mobile medical service trips. Table 1 provides detailed information about the three driving routes, highlighting the diverse road conditions encountered during transportation and their potential impact on the stability of dental equipment.

**Table 1:** Detailed information regarding the transportation routes.

Driving route	Time consumed (h:m)	Actual route length (km)	Average speed (km/h)	Highest speed (km/h)	Lowest altitude (m)	Highest altitude (m)	Total ascend (m)	Total descend (m)
100 km route	3:59	99.1	24.9	62.7	20	798	1480	1476
200 km route	4:47	221.9	46.4	106	18	301	1801	1804
300 km route	8:22	310	37	92.2	19	552	2710	2714

Before each departure, the tire pressure was consistently checked and maintained at 40 psi. While driving, the Global Positioning System (GPS) information was recorded using the MyRoute app (MyRouteApp B.V., Zoetermeer, Netherlands). The vibration and the shock that the dental milling machine endured during the transportation process were also recorded on the MSR165 data logger (MSR Electronics GmbH, Seuzach, Switzerland), which was attached to the top of the CNC milling machine. The recording frequency is one record per second.

After completing each route, four crowns were milled to evaluate the impact of transportation on milling accuracy. The STL file of a single lower molar crown, initially designed as the master digital model using Exocad Dental CAD software (Exocad GmbH, Darmstadt, Germany), was imported into Millbox software (CIMSystem, Cinisello Balsamo, Italy) for further modifications. In Millbox, a connector was added to the crown's mesial-lingual side, and layout adjustments and milling strategy selections were performed. Wet-milling was conducted using Amber® Mill Direct lithium disilicate blocks (HASSbio America, Fairfax, VA, USA) in A2 shade, with Diamond milling bits (0.6, 1.0, and 2.6 mm) (Toshin).

To test the accuracy of the crowns and their potential deviations due to the transportation routes, the milled crowns were scanned using a Medit T710 scanner (Medit Corp., Seoul, Korea). The scanned STL files were compared with the master digital model as the reference using Geomagic™ software (3D Systems, Rock Hill, SC, USA). The software applied the iterative closest point algorithm to align the reference and test files, achieving a best-fit alignment with a maximum of 20 iterations and a 0.5 mm maximum average deviation. A 3D comparison was performed with a 100 % specimen ratio, shortest projection direction, deviation limits of +0.5 mm and −0.5 mm, and a specific tolerance of ±50 μm, presented as color mapping. The occlusal surface, inner surface, and crown margin were analyzed separately to detect deviations in these critical areas.

The RMS, the deviation of the milled crowns from the master digital model, and the percentage of the surface area with less than ±50 μm deviation were expressed as the mean and standard deviation. An analysis of variance (ANOVA) was performed using software program SPSS® version 24.0 (IBM SPSS Inc., Chicago, IL, USA) with a significance level (α) of 0.05.

## Results

**Figure 3:**
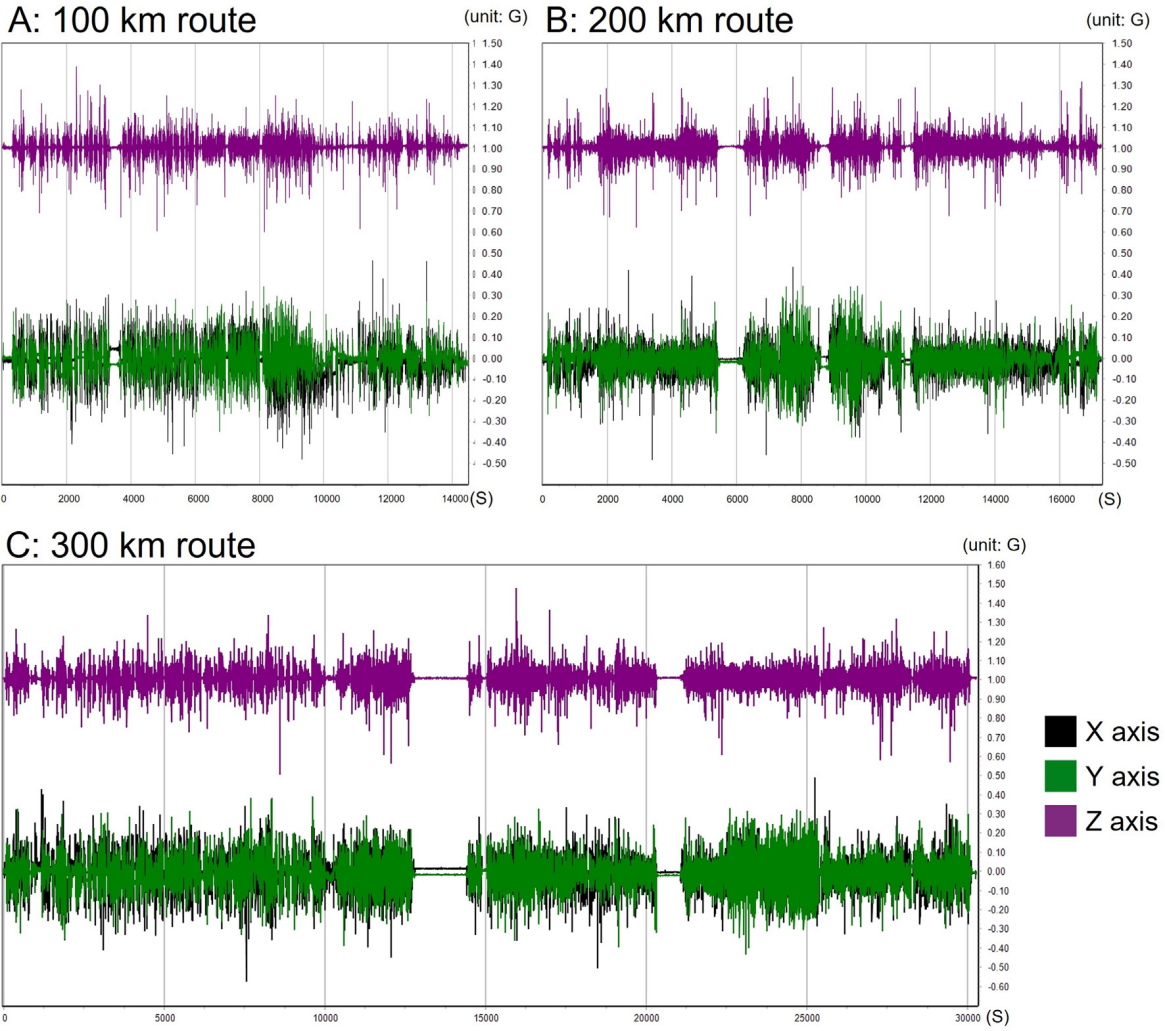
Vibration records for the routes: (A) 100 km, (B) 200 km, and (C) 300 km. The purple spectrum represents vibrations in the X direction, while the green spectrum represents vibrations in the Y direction. The black spectrum (overlapped with the green spectrum) also represents vibrations in the X direction. The X-axis indicates time in seconds after departure, and the Y-axis represents acceleration detected, measured in G.

MSR data logger recorded the vibration of the milling machine during transportation of the dental milling machine (Fig. 3). Basically, detectable vibrations were observed throughout three routes. Flat regions of the spectra were the period of time when the driver took a brake or had meals. The Z axis had a base line at 1 G while the X and Y axis had base lines at 0. The 1 G baseline at Z axis is because of the earth gravity. Most of the vibration along X and Y axes for all three routes ranged between ±0.2 G, while most of the vibration along Z axis for all three routes ranged between ±0.15 G. The vibration along Z axis is generally smaller than vibration along X and Y axes.

The numerical integration of the spectra over time (Table 2) reveals that the Y-axis G∗S value for the 300 km group is nearly equal to the combined total of the 100 km and 200 km groups. Meanwhile, the X-axis G*S* value for the 200 km group is slightly lower than that of the 100 km group. This difference is attributed to Route 2, which primarily consists of highways and expressways, resulting in fewer sudden accelerations or decelerations during transport.

**Table 2:** The numerical integration of the vibration spectra of three different routes along the X, Y, and X axes.

	Integration of X axis	Integration of Y axis	Integration of Z axis
100 km route	−211.516 G∗S	−74.252 G∗S	14548.746 G∗S
200 km route	−197.804 G∗S	−167.450 G∗S	17400.826 G∗S
300 km route	−271.586 G∗S	−328.887 G∗S	30493.993 G∗S

**Figure 4:**
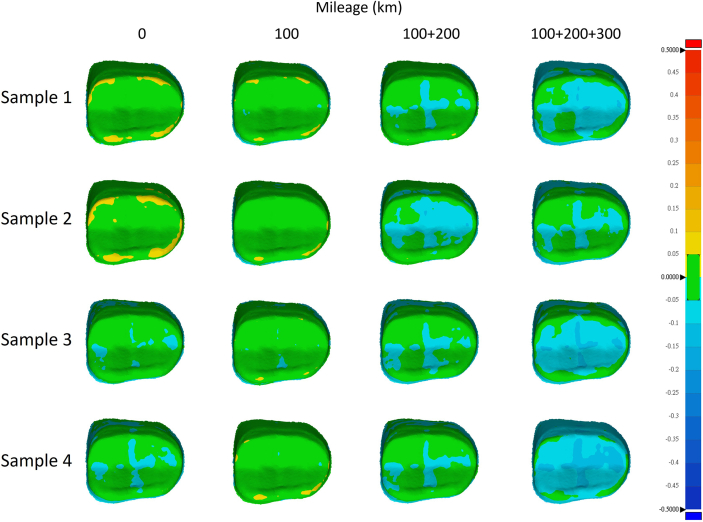
Heat maps generated by superimposing the inner surfaces of the 3D digital models obtained from scans of the milled blocks onto the 3D digital models of the originally designed crown. Areas with a difference of 50 μm are highlighted in green.

**Figure 5:**
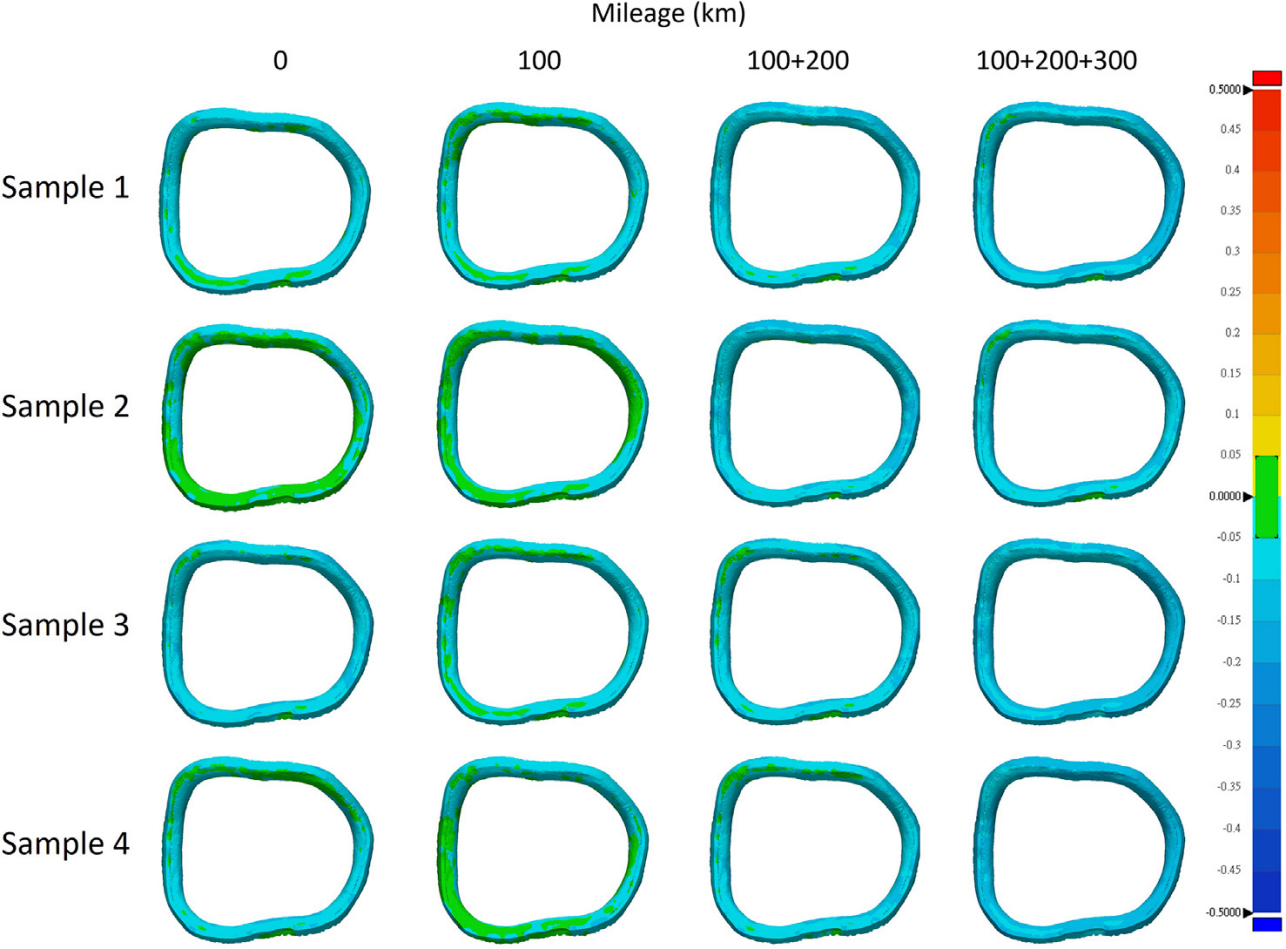
Heat maps generated by superimposing the crown margins of the 3D digital models obtained from scans of the milled blocks onto the 3D digital models of the originally designed crown. Areas with a difference of 50 μm are highlighted in green.

**Figure 6:**
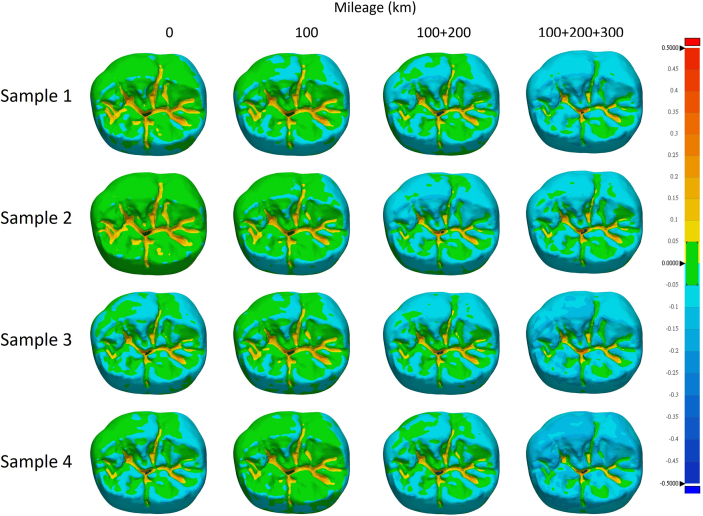
Heat maps generated by superimposing the occlusal surfaces of the 3D digital models obtained from scans of the milled blocks onto the 3D digital models of the originally designed crown. Areas with a difference of 50 μm are highlighted in green.

The accuracies of the milled crowns were represented as color mapping of the deviations of the milled crowns from the master digital model (Figure 5, Figure 6). Green area represented deviations ranging between −50 μm and 50 μm, while other colors represented deviations larger than 50 μm or smaller than −50 μm. In this test, the cumulative mileage of 100 km, 100 + 200 km, and 100 + 200+300 km were used for grouping. The 0 km group was used as a control. Figure 4, Figure 5, Figure 6 demonstrate that as cumulative millage increased, the areas on the occlusal surface, inner surface, and crown margin with deviations exceeding +50 μm or falling below −50μm also expanded. This trend can be clearly observed in the 100 + 200+300 km group.

**Figure 7:**
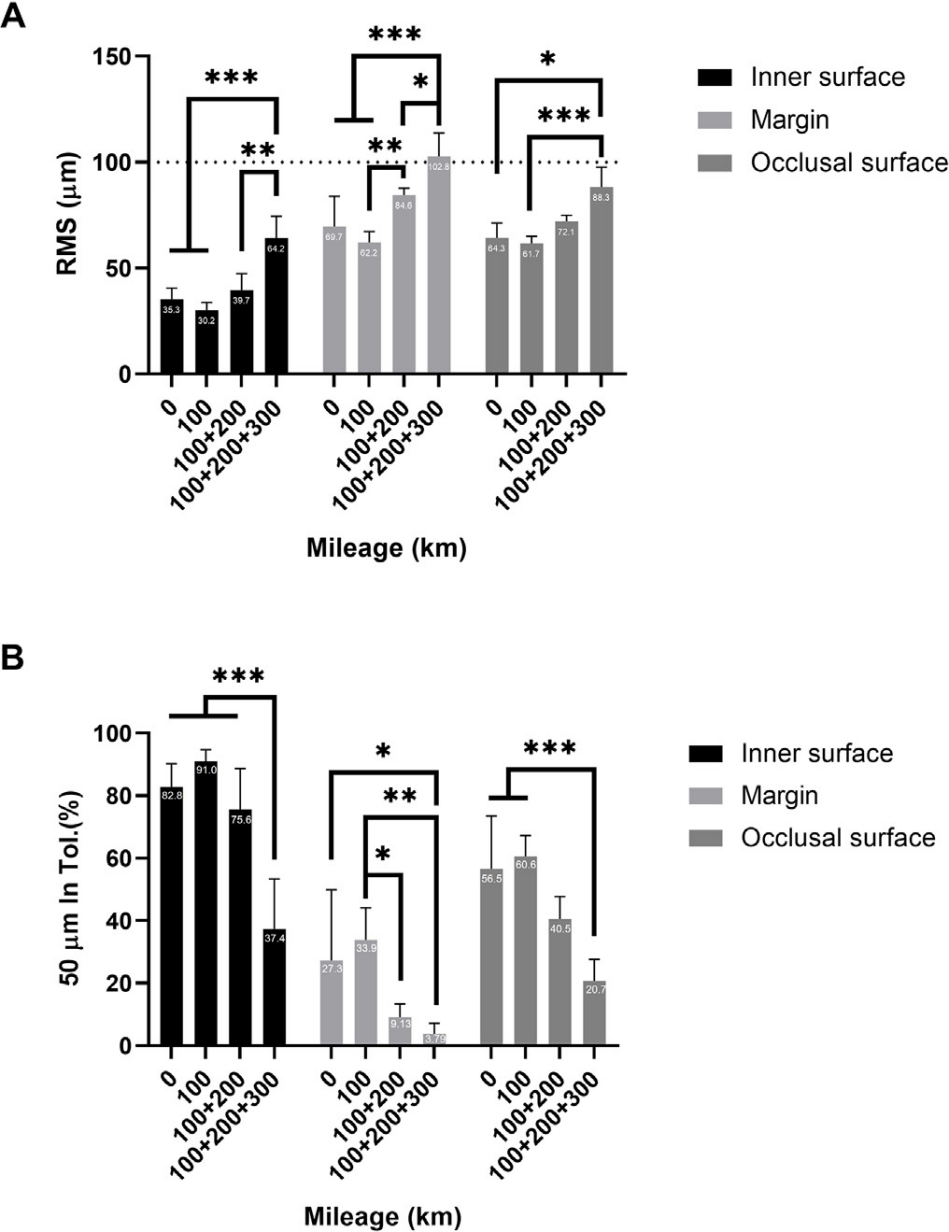
(A) Root mean square (RMS) deviation of the milled crowns compared to the originally designed STL file. The dashed line represents a loose standard for crown margin fit. (B) Percentage of the surface area of the milled crowns with deviations within the ±50 μm range. After 100 km, 200 km, and 300 km of transportation, the surface area with deviations within the ±50 μm range significantly decreased compared to the control. (∗*P* < 0.05, ∗∗*P* < 0.01, ∗∗∗*P* < 0.001).

The results from Figure 4, Figure 5, Figure 6 were converted to a bar graph with statistical analysis in Fig. 7. Fig. 7A showed that after 100 + 200+300 km of transportation, the occlusal surface, inner surface, and crown margin of the milled crowns had RMS errors significantly higher than the control and other groups. In contrast, the RMS errors of the 100 km and 100 + 200 km groups did not significantly differ from the control. A dashed line was drawn to represent the value of the 100 μm, which was considered to be the loose standard of a crown margin fit.^3^

For the inner surface and occlusal surface, the 100 + 200+300 km group exhibited a statistically significantly lower percentage of surface areas with deviations under 50 μm (Fig. 7B) compared to other groups, while the 100 km and 100 + 200 km groups showed results comparable to the control. For the crown margin, the 100 + 200 km and 100 + 200+300 km groups had significantly lower percentages of surface areas with deviations under 50 μm than both the control and the 100 km groups.

## Discussion

In this study, Amber mill direct blocks was chosen for milling. The main reason is that it does not require sintering before delivery. Therefore, it can save the precious time for the circuit dental service. Amber Mill Direct is a lithium disilicate-based ceramic material with a strength of around 250 MPa and requires no sintering process before use.^4^ Its indication include inlay, onlay, veneer, anterior single crown and posterior single crown with occlusal wall thicker than 2 mm. Also, this material has been reported extensively in literature.^4^, ^5^, ^6^ Because of the reasons mentioned above, this material is the material of choice for this study.

The MSR recorder can record the vibration or shocking of the dental milling machine during transportation. Shock and vibration are two distinct mechanical phenomena despite both involving oscillatory motion. Shock refers to a rapid, transient force or disturbance that typically generates a sudden change in momentum and creates a high-amplitude impact, such as collisions and abrupt mechanical failures. Vibration, on the other hand, denotes a periodic motion that is often characterized by its frequency, amplitude, and duration. It can be caused by various factors, e.g., driving on uneven surfaces and external environmental forces. Unlike shock, which exhibits a rapid change, vibration tends to have a repetitive nature, typically occurring over longer periods of time. Shock loads, due to their impulsive nature, can lead to immediate failure, yielding, or catastrophic structural damage, while vibration may induce fatigue over time, potentially compromising structural integrity. The only two conditions for a dental CNC milling machine that will be subject to shock force include traffic accidents and falling apart from its base. Both conditions will result in the machine's immediate failure. Therefore, vibration mode was chosen for MSR165 data logger.

Dental CNC milling machines are sophisticated tools used for the precision machining of dental appliances. These machines operate by removing material from a workpiece using rotary cutters which are controlled by computer programs. A dental CNC milling machine is composed of so many movable parts, so that its accuracy is the combinatorial results of the accuracy of its moving parts and the strength of the whole machine structure.

Transportation vibrations can significantly impact the accuracy of CNC milling machines, particularly when these machines are transported over long distances or subjected to rough handling. For example, a rigid frame is essential for minimizing vibrations during operation; any deformation may affect the milling accuracy.^7^ Transportation vibrations may loosen joints or structural integrity of the milling machine. The block holder's relative position to the milling bits is crucial for accurate grinding of the ceramic blocks. Shipping vibrations can cause the displacement of the block holders during transportation, affecting their ability to move to the designated position during machine operation. The feed drive system, sometimes called X-Y-Z stages, is vibration-sensitive. Shipping vibrations can induce wear, misalignment or minor displacement in ball screws and linear guides of the feed drive system and thus compromise their performance.^8^

Despite so many factors that can affect the accuracy of the dental CNC milling machine,^9^, ^10^, ^11^ we still decided to mount the milling machine on the load compartment without any vibration-damping device in between, nor did we wrap the milling machine with wrapping materials. The decision is based on the fact that the Tosin idensol 5XG five-axis dental milling machine had a strong framework structure. If it loses milling accuracy during transportation, the most likely cause is the displacement of the major moving parts: the block holder and the feed-drive system.

The results showed that as the cumulative millage increased, the areas of occlusal surface, inner surface and crown margin that had deviations larger than 50 μm or smaller than −50μm increased. This deviation might be caused by the deviation of the block holder and the feed-drive system. More evidence to prove the statement should be performed in future studies.

Fit accuracy is paramount in ensuring that a ceramic crown properly aligns with the prepared tooth structure. The marginal fit—the gap between the crown and the tooth margin—should ideally be less than 100 μm to prevent complications such as microleakage and secondary caries.^3^

In this study, the milled crowns were scanned and compared with the digital master model using Geomagic. The control, 100 km and 100 + 200 km group had the RMS deviation of the crown margin were 70 ± 14, 62 ± 5, and 85 ± 3 μm, respectively. These values were lower than 100 μm standard. Therefore, we can assume that within 300 km of transportation, dental CNC milling can still mill ceramic crowns with clinically acceptable marginal fit without requiring thorough machine calibration and inspection.

Regarding the inner surface fit, the control, 100 km, and 100 + 200 km groups showed inner surface deviations of less than 40 μm, while the 100 + 200+300 km group had a deviation of 64.2 μm. Although the inner surface fit can be adjusted by incorporating a cement space during the design process, this increased deviation highlights potential challenges in maintaining precise fit over extended distances.

The mobile medical unit offers a valuable solution to overcoming geographic barriers to dental care, particularly in rural and remote areas. However, its limitations include the need for specialized training to effectively use digital equipment, along with high initial investment and ongoing costs for maintenance and upgrades. Additionally, efficient treatment can be challenging without printed models, as operator errors may lead to inaccuracies, highlighting the need for experienced dental technicians. There are no regulations governing the involvement of dental technicians in rural medical services, unlike dentists, who can report and provide support in such settings. Despite these challenges, circuit dental services play a crucial role in community health by making dental care more equitable and accessible to individuals regardless of location.

The mobile digital dental lab enhances these services by providing advanced procedures directly on-site, such as CNC milling for crowns and dentures. This innovation offers timely and efficient care, benefiting patients from immediate treatment. To the authors' knowledge, this mobile digital dental lab is the first of its kind in the world. This study demonstrated that within a 300 km transportation distance, the dental milling machine can be operated immediately upon arrival, successfully delivering single crowns that meet clinical requirements. Future studies will focus on the accuracy of CNC-milled dentures and long-span bridges, further expanding the potential applications of mobile dental care.

## Declaration of competing interest

The authors have no conflicts of interest relevant to this article.
